# Preemptive local analgesia at vaginal hysterectomy: a systematic review

**DOI:** 10.1007/s00192-021-04999-1

**Published:** 2021-12-06

**Authors:** Nadja Taumberger, Anna-Maria Schütz, Klaus Jeitler, Andrea Siebenhofer, Holger Simonis, Helmar Bornemann-Cimenti, Rene Laky, Karl Tamussino

**Affiliations:** 1 Department of Obstetrics & Gynecology, Medical University of Graz, Auenbruggerplatz 14, 8036 Graz, Austria; 2 Institute of General Practice and Evidence-based Health Services Research, Medical University of Graz, Graz, Austria; 3 Institute for General Practice, Goethe University Frankfurt am Main, Frankfurt, Germany; 4 Department of Anesthesiology, Emergency Medicine and Critical Care, Medical University of Graz, Graz, Austria

**Keywords:** Postoperative pain, Local preemptive analgesia, Vaginal hysterectomy

## Abstract

**Introduction and hypothesis:**

We conducted a systematic review of the effectiveness of local preemptive analgesia for postoperative pain control in women undergoing vaginal hysterectomy.

**Methods:**

MEDLINE, EMBASE, the Cochrane Central Register of Controlled Trials and the Cochrane Database of Systematic Reviews were searched systematically to identify eligible studies published through September 25, 2019. Only randomized controlled trials and systematic reviews addressing local preemptive analgesia compared to placebo at vaginal hysterectomy were considered. Data were extracted by two independent reviewers. Results were compared, and disagreement was resolved by discussion. Forty-seven studies met inclusion criteria for full-text review. Four RCTs, including a total of 197 patients, and two SRs were included in the review.

**Results:**

Preemptive local analgesia reduced postoperative pain scores up to 6 h and postoperative opioid requirements in the first 24 h after surgery.

**Conclusion:**

Preemptive local analgesia at vaginal hysterectomy results in less postoperative pain and less postoperative opioid consumption.

## Introduction

Hysterectomy for benign indications is one of the most common operations in gynecology. Multiple guidelines and reviews favor the vaginal approach for benign hysterectomy, if feasible [1–5]. In German-speaking countries, vaginal hysterectomy is the most common approach to hysterectomy, with about half of all benign hysterectomies done vaginally [1, 6].

Many Enhanced Recovery After Surgery (ERAS) protocols in gynecology recommend multimodal analgesia using different agents addressing different pathways to reduce intra- and postoperative opioid requirements, speed recovery and reduce complications [2, 3, 7]. The reduction of opioid requirements is of particular significance considering the potential for misuse of these agents [8–10], their side effects and higher costs for the health care system [7]. Recently attempts have been made to improve multimodal perioperative analgesia [8, 9, 11–19]. Due to the opioid crisis, there is high interest in reducing perioperative opioid use. Preemptive analgesia is a part of this concept and denotes all analgesia given before the start of surgery, i.e., before any painful stimulus to the body [20].

We performed this systematic review (SR) because of the clinical relevance of postoperative pain control in a frequently performed procedure.

The primary aim was to systematically review the literature on vaginal hysterectomy with any form of local preemptive analgesia according to postoperative pain reduction. Secondary outcomes were defined as postoperative opioid requirements, readmission rates, perioperative pain management and quality of life measured by validated questionnaires as well as opioid-related side effects.

## Materials and methods

The systematic review was restricted to the use of local preemptive analgesia in vaginal hysterectomy; other modes of hysterectomy and other forms of analgesia interventions were excluded. The protocol was registered at PROSPERO and is available under https://www.crd.york.ac.uk/prospero/display_record.php?ID=CRD42020144709. PRISMA guidelines were followed [21]. No approval was needed from the institutional review board because of the study design.

### Data sources and search strategy

Two gynecologists systematically searched MEDLINE (1946 to present), EMBASE (1974 to present), the Cochrane Central Register of Controlled Trials (CCTR) and the Cochrane Database of Systematic Reviews (CDSR) to identify relevant randomized controlled trials (RCT) and SR. Subject headings and keywords for vaginal hysterectomy were suitably combined with those for local preemptive analgesia or local anesthetics as well as filters for randomized controlled trials or systematic reviews. No restrictions for the date of publication were made, and all full text articles that were published in either English or German were included while those written in other languages were excluded. Reference lists of eligible studies and review articles were included in the search.

Two reviewers (N.T. and A.-M. S.) screened the identified abstracts and removed duplicate entries. Subsequently, all full texts of potentially relevant abstracts were retrieved and screened in the same way. Any discrepancies were resolved by consensus. The screening process and its results were documented in a spreadsheet.

The two reviewers used prespecified extraction templates to independently extract the data. Extracted data included information on the study type and methodology, country/place of the study, inclusion and exclusion criteria, participant demographics, number of participants and measured outcomes and effects. Disagreement was resolved by discussion between the reviewers.

### Study selection

The review focused on RCTs and SRs of local preemptive analgesia given prior to vaginal hysterectomy for all indications with the goal of reducing postoperative pain, peri- and postoperative opioid use as well as readmission rates. The search was through 25 September 2019. The intervention had to be compared to another regime or placebo. We excluded laparoscopic or laparoscopically assisted vaginal hysterectomies. For more homogeneous data we also excluded systemic interventions and spinal interventions. Studies including vaginal hysterectomy done for prolapse were included. No restrictions were made on the basis of sample size, country or date of publication.

### Data extraction and quality assessment

The quality of the RCTs was assessed with the current version of the Cochrane Risk of Bias Tool [22], which comprises domains such as the randomization process, deviations from intended interventions, missing outcome data, measurement of the outcome and selection of the reported result. Because we included only local interventions compared to placebo or no local treatment, we did not need to categorize the different interventions according to their location, but we did categorize them according to their comparison to placebo or no local treatment, participant characteristics and intervention details.

## Results

**Fig. 1:**
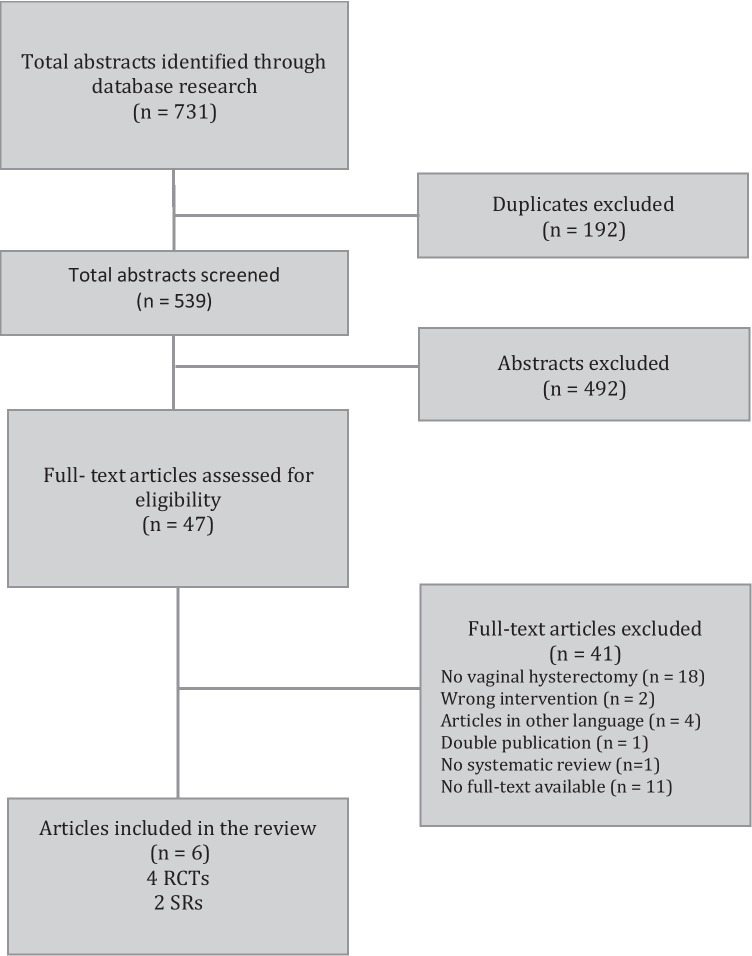
Literature selection process

A total of 731 abstracts were identified, and after removal of the duplicates, 539 were screened and 47 full-text manuscripts were selected for further evaluation. As our review focused strictly on preemptive local analgesia in vaginal hysterectomy, we identified 4 RCTs with a total of 197 patients and 2 SRs for inclusion in the SR (Fig. 1). All four RCTs compared local preemptive analgesia with placebo using different local anesthetics.

One study excluded vaginal hysterectomies done for prolapse [23], one study included these procedures [24], one study included only women with prolapse [25], and one is unclear on this question [26].

### Applied local anesthetic

Two of the included studies compared 30 ml of 0.5% of ropivacaine vs. placebo [24, 25], and the other two compared 20 ml of 0.5% bupivacaine combined with 1:200,000 epinephrine vs. placebo infiltration [23, 26].

### Main outcome

Tables 1 and 2 summarize the significant outcomes and study characteristics of the four studies.

**Table 1:** Study characteristics and significant results"-->

Authors	Study type	N	Surgery	Medication	Injection technique	Comparison	Baseline medication	VAS or NRS pain score	Postoperative opioid consumption (24 h)	Conclusion
O’Neal et al. [26]	Randomized, double-blind, placebo-controlled trial	20	VH	20 ml 0.5% bupivacaine with 1:200,000 epinephrine	5 ml was injected at the 3, 5, 7 and 9 o’clock positions of the cervicovaginal junction	20 ml 0.5% saline with 1:200,000 epinephrine	No information regarding preoperative pain medication or anesthesia protocol. Morphine patient-controlled analgesia (PCA) at the post- anesthesia care unit (PACU). Ketorolac for breakthrough pain	1.7 vs. 3.3 (4 h) 1.8 vs. 3.6 (6 h) *p* = 0.003	22 mg vs. 37 mg *p* = 0.01	Significant decrease in pain scores and reduction in morphine requirements in the first 24 h after surgery
Long et al. [23]	Randomized, double-blind, placebo-controlled trial	90	VH	20 ml 0.5% bupivacaine with 1:200,000 epinephrine	10 ml (5 ml into each uterosacral ligament) in a paracervical block fashion using a 22-gauge needle 4 min prior to initial incision. Additional 10 ml (5 ml into each uterosacral ligament) was injected into the more proximal uterosacral ligaments just prior to placement of McCall culdoplasty sutures	20 ml 0.5% saline with 1:200,000 epinephrine	Routine preoperative administration of famotidine and dexamethasone General anesthesia: fentanyl 2 μg/kg and propofol 2 mg/kg, nitrous oxide 70%, sevoflurane and additional fentanyl as needed. At conclusion, either ketorolac and/or ondansetron was administered Postoperatively, patients were given narcotics, ketorolac and antiemetics as needed	2.5 (3.1) vs. 4.4 (3.0) (30 Min) *p* = 0.003 2.4 (2.4) vs. 3.6 (2.0) (3 h) *p* = 0.02	16 mg vs. 23 mg *p* = 0.009	Significant decrease in pain scores after 30 min and 3 h after surgery Significant reduction of opioid use over 24 h
Hristovs.ka et al. [24]	Randomized, double-blind, placebo-controlled trial	37	VH	30 ml ropivacaine 0.5%	30 ml was injected in a systematic fashion as a modified paracervical block. First, 5 ml was injected through the vaginal fornices at 02.00, 04.00, 06.00, 08.00, 10.00 and 12.00 h at 2 cm depth while the needle was retracted	30 ml saline	Routine preoperative administration of celecoxib 400 mg and slow-release paracetamol 2 g General anesthesia: propofol 2–3 mg/kg and remifentanil 0.5 lg/kg/min, continuous infusion of propofol 10 mg/ml, 4–6 mg/kg/h and remifentanil 2 mg, 0.25–0.5 lg/kg/min. PACU: Sufentanil 5 lg until the VAS was ≤ 3 Ward: celecoxib 200 mg/12 h and slow-release paracetamol 2 g/12 h as well as oral oxynorm 5 mg on request if the VAS was ≥ 5	Resting: 10 (0–35) vs. 60 (30–90) (1 h) 15 (0–52) vs. 45 (17–80) (4 h) 19 (4–45) vs. 40 (8–53) (8 h) *p* ≤ 0.001–0.01 Coughing: 10 (0–45) vs. 70 (30–96) (1 h) *p* = 0.001 18 (0–77) vs. 50 (18–82) (4 h) p = 0.003	10 mg vs. 25 mg *p* ≤ 0.007	Significant decrease in pain scores after 1 h, 4 h and 8 h at rest and 1 h and 4 h at coughing. Significant reduction in opioid consumption over 24 h
Athanasiou et al. [25]	Randomized, double-blind, placebo-controlled trial	59	VH	30 ml ropivacaine 0.5%	Infiltration of 30 ml ropivacaine 0.5% (5 ml in round ligament and 5 ml in uterosacral ligament bilaterally, and 10 ml in perineal body, while the placebo group received an infiltration of 30 ml placebo solution in the same fashion)	30 ml saline	CSE block was performed with 2.5 ml ropivacaine 0.75% plus 15 mg fentanyl Preoperative administration of antibiotics, metoclopramide 10 mg, ranitidine 100 mg, diclofenac 75 mg and paracetamol 1 g Postoperative PCA containing 40 mg morphine (0.5 mg/ml) plus 8 mg ondansetron (0.1 mg/ml) was used. All participants received metoclopramide 10 mg/12 h i. v., ranitidine 100 mg/12 h i.v., paracetamol 1 g/8 h i.v. and diclofenac suppository 75 mg/12 h. Additionally, ondasetron 4 mg i.v. for nausea and vomiting	Resting: 0.5 (0.1–7.2) vs. 1.1 (0.2–9.3) (2 h) *p* = 0.007 1.3 (0.1–5.1) vs. 3.1 (0.1–9.8) (4 h) p = 0.02 Coughing: 0.9 (0.1–8.9) vs. 1.9 (0.1–10) (2 h) *p* = 0.03 1.6 (0.1–4.7) vs. 3.2 (0.3–9.6) (4 h) p = 0.009	4 mg vs. 7 mg p = 0.02	Significant decrease in pain scores 2 h and 4 h at rest and 2 h and 4 h at coughing Significant reduction of opioid consumption over 24 h after surgery

**Table 2:** All pain scores with significant results in bold

	Athanasiou et al.	Hristovs.ka et al.	Long et al.	O’Neal et al.
	25 (IG*) vs. 25 (CG*)	20 (IG) vs. 17 (CG)	45 (IG) vs. 45 (CG)	9 (IG) vs. 11 (CG)
Oost OP time (h)	Pain at rest (median VAS)	Pain at rest (median VAS)	Pain (mean VAS)	Verbal analog pain score
**0.5**	–	–	**2.5 vs. 4.4**	
**1**	–	**10 vs. 60**	–	5.3 vs. 4.8
**2**	**0.5 vs. 1.1**	20 vs. 35	–	3.4 vs. 4.9
**3**	–	–	**2.4 vs. 3.6**	3.1 vs. 4.6
**4**	**1.3 vs. 3.1**	15 vs. 45	–	**1.7 vs. 3.3**
**6**	–	–	–	**1.8 vs. 3.6**
**8**	1.3 vs. 2.6	**19 vs. 40**	–	–
**12**	–	24 vs. 29	3.0 vs. 2.7	–
**24**	0.5 vs. 0.6	2 vs. 2	2.2 vs. 2.0	1.4 vs. 1.7
**32**	–	No data	–	–
	Pain during cough (median VAS)	Pain during cough (median VAS)	–	–
**1**	–	**10 vs. 70**	–	–
**2**	**0.9 vs. 1.9**	22 vs. 35	–	–
**4**	**1.6 vs. 3.2**	**18 vs. 50**	–	–
**8**	1.7 vs. 4	20 vs. 46	–	–
**12**	–	29 vs. 38	–	–
**24**	0.5 vs. 1	19 vs. 20	–	–
**32**		No data	–	–
	**VAS ≥ 4 (n/N)**	–	**VAS = 0 (n/N)**	–
**0.5**	–	–	25/45 vs. 11/45	–
**2**	1/25 vs. 8/25	–	–	–
**3**	–	–	14/45 vs. 6/45	–
**4**	4/25 vs. 11/25	–	–	–
**8**	3/25 vs. 10/25	–	–	–
**12**	–	–	12/44 vs. 13/45	–
**24**	2/25 vs. 1/25	–	16/44 vs. 14/45	–

The primary outcomes of all four studies were postoperative pain measured with either the visual analogue scale (VAS) or a verbal analogue pain score from 0 to 10 at different predefined time points between 30 min (min) and 32 h (h) after surgery. Two studies evaluated postoperative pain at rest, one while resting and during coughing and one defining the primary outcome of postoperative pain as pain intensity while coughing.

Both studies which evaluated pain at rest showed a significant reduction in pain for 30 min up to 6 h after surgery. Pain during coughing was also significantly reduced at 1 and 4 h postoperatively in the treatment groups in the two studies that assessed this (Table 2).

### Other outcomes

Our predefined secondary outcomes in the four studies included blood loss, length of hospitalization, adverse events, duration of surgery, postoperative nausea and vomiting, and time to first mobilization. These outcomes did not differ between the two groups with or without preemptive analgesia. None of the four RCTs measured quality of life (QoL), readmission rate or perioperative care, so no results to these predefined secondary end points of our protocol can be reported.

None of the four included RCTs reported any adverse events regarding the use of preemptive local anesthesia.

The studies which defined pain at rest or during movement or pain at other evaluated time points as secondary outcomes found also a significant decrease in pain between 1 and 8 h. One secondary outcome all four studies had in common was postoperative morphine consumption. Although the results regarding opioid use in post-anesthesia care were different in two studies, all studies showed a significant reduction in morphine-controlled patient analgesia and/or overall opioid requirements in the treatment group in the first 24 h.

Using the Cochrane Risk of Bias Tool [22], we assessed three studies [23–25] as having a low risk of bias with clear methods. One of the RCTs [26] lacked specific descriptive statistical analysis and provided no information on confidence intervals used whether mean or median values were reported. This study summarized all pain scores in one figure with only the greatest difference in pain scores appearing after 4 and 6 h. After analyzing the data from the figure, we assumed that the columns showed the mean of the pain scores.

#### Use of statistics

A meta-analysis was planned but due to the heterogeneity of time points and conditions under which the outcomes were measured, the results of the studies were analyzed descriptively.

## Discussion

Our systematic review of preemptive local analgesia at vaginal hysterectomy yielded four RCTs with a total of 197 randomized patients [23–26]. To our knowledge, this is the first SR of this specific issue.

### Main findings

All four RCTs showed a significant decrease in postoperative pain with the use of preemptive analgesia at different measurement points up to 8 h after surgery and a decrease in morphine use over the first 24 h after surgery. However, the effect on postoperative pain reduction is only seen up to 8 h postoperatively, which means that the effect is only measurable on the day of surgery. This is consistent with the half-life of widely used local anesthetic agents. This explains the lack of difference in length of hospital stay [23, 24]. Also, no significant difference was shown in the adverse events of opioid consumption such as nausea, vomiting or sedation [24, 25], which is probably explainable because of the rather small sample size.

Regarding postoperative pain and opioid consumption, the effects were statistically significant and clinically measurable, but the total number of patients investigated was small. However, besides the pain scores reported by Hristovs.ka et al., all postoperative pain score means and medians were under 45 mm on the VAS scale.

There are different VAS cutoffs for mild, moderate and severe pain for the VAS scale ranging from 30, 70 and 100 mm [27] to 44, 74 and 100 mm [28]. Based on guideline recommendations and studies using patient controlled analgesia, a VAS of ≤ 33 mm is considered acceptable pain right after surgery [27]. This means that a great part of the patient population had good postoperative pain control anyway—with or without preemptive analgesia.

Athanasiou et al. [25] studied only patients with prolapse surgery and used combined spinal-epidural block (CSE) instead of general anesthesia. They evaluated two primary end points: postoperative pain scores and the number of patients who had moderate or severe pain, defined as a VAS score ≥ 4 on a 10-cm VAS scale. They showed a significant decrease in the number of patients with higher pain scores up to 8 h after surgery accompanied by significantly less opioid consumption up to 24 h after surgery. The duration of 8 h is explained by the duration of the sensory block of ropivacaine, which is approximately 6–10 h [24, 29]. Although we saw a trend in favor of fewer patients reporting opioid side effects, this was not significant and was likely due to standard use of antiemetics and systemic NSAIDs [25]. For example, Hristovs.ka et al. [24] found that the reduction of postoperative opioid use did not lead to a decline in opioid side effects. Postoperative pain scores were also significantly lower in the treatment group up to 8 h, which is also in line with the 8–13 h duration of bupivacaine [29]. The main difference between this RCT and the other three is that the pain scores in this study were higher than in the others, for reasons which are unclear [24]. The reason for this was not obvious, but because of the similar pain scores of the other three studies with a patient number of 160, assumptions can be made that either there was another surgical approach or the perioperative pain management differed from that of the other studies.

The largest study in this review [23] randomized 90 patients and used 20 ml 0.5% bupivacaine with 1:200,000 epinephrine. They found a decline in pain scores up to 3 h after surgery as well as a reduction in opioid consumption.

The earliest study in our review, published in 2003 [26], has the highest risk of bias indicated by the Cochrane Risk of Bias Tool [22]. The pain results are similar to those in the other studies but statistical methods, perioperative analgesia, the anesthetic protocol and results are not described clearly.

There has been much debate about what amount of VAS change is clinically important. A prospective observational study enrolling 224 patients suggested the minimal clinically important difference (MCID) to be 10 in the postoperative setting measured by the VAS scale [27]. However, because of the heterogeneity of surgical procedures, preexisting conditions and individual patients, we have no validated and evidence-based recommendation on the MCID in the postoperative setting [30].

The postoperative pain scores in the present review indicate that patients undergoing vaginal hysterectomy have good postoperative pain control even without preemptive analgesia. With medians and means of 3.1, 4.5, 3.6 and 3.3 points between 3 and 4 h after surgery, most of the patients had postoperative pain scores that can be considered acceptable [27]. Nevertheless, the reported medians and means of 1.3, 1.5, 2.4 and 1.7 of the treatment group were significantly lower, and the suggested MCID of 1 point on the VAS scale was reached in all studies.

All four trials in this review found reduced postoperative opioid requirements during the first 24 h after surgery [23–26]. Many concepts have been implemented in the last few years to reduce postoperative opioid needs in gynecologic patients, including a shared decision-making model [14], change in discharge regimes in minimal invasive surgeries [13] and a quality improvement intervention protocol [12]. Systemic approaches to multimodal analgesia have included systemic administration of acetaminophen and anti-inflammatory drugs and gabapentin [31–37]. Our results regarding the benefits of a paracervical block before vaginal hysterectomy are in line with those of the other SRs [35, 38].

### Strengths

This is a systematic review of a simple intervention in a frequently performed operation with a clear result. An inexpensive and simple intervention – i.e., preoperative infiltration with a local anesthetic agent, improves patient outcomes.

### Limitations

The results of our SR are limited by the studies available. Our review yielded four RCTS with < 200 patients overall. A meta-analysis was not possible because of heterogeneity of end points as well as the use of different local analgesics and the small number of studies included. Also, a sub-analysis according to the indication for vaginal hysterectomy was not possible because of heterogeneity.

### Conclusion

The data from four RCTs, with three of them being of good quality, indicate that local preemptive analgesia in the form of a paracervical block is a simple procedure, which results in lower postoperative pain scores and opioid consumption of patients undergoing vaginal hysterectomy. Another systematic review has already described the need for further evidence in minimally invasive hysterectomies [39]. National and international guidelines recommend the vaginal approach [1, 2, 4, 5], and according to fast track pathways [40] and ERAS protocols [3, 7,7,^37^, ^41^], multimodal analgesia protocols are recommended to reduce postoperative opioid consumption and improve patient recovery.

Given its easy implementation and low cost, local preemptive analgesia in vaginal hysterectomy is a simple but effective procedure to improve postoperative pain control.

None of these studies used a long-acting, liposomal-bound agent, and this might be a topic for future research. Only one RCT has compared liposomal bupivacaine vs. placebo in posterior vaginal wall surgery and found no significant decrease in postoperative pain or narcotic medication [42].
